# Mental fatigue in stress-related exhaustion disorder: Structural brain correlates, clinical characteristics and relations with cognitive functioning

**DOI:** 10.1016/j.nicl.2020.102337

**Published:** 2020-07-03

**Authors:** Hanna Malmberg Gavelin, Anna Stigsdotter Neely, Tora Dunås, Therese Eskilsson, Lisbeth Slunga Järvholm, Carl-Johan Boraxbekk

**Affiliations:** a Academic Unit for Psychiatry of Old Age, University of Melbourne, Melbourne, Australia; b Department of Psychology, Umeå University, Umeå, Sweden; c Umeå Centre for Functional Brain Imaging (UFBI), Umeå University, Umeå, Sweden; d Department of Social and Psychological Studies, Karlstad University, Karlstad, Sweden; e Centre for Demographic and Aging Research (CEDAR), Umeå University, Umeå, Sweden; f Department of Community Medicine and Rehabilitation, Physiotherapy, Umeå University, Umeå, Sweden; g Department of Public Health and Clinical Medicine, Sustainable Health, Umeå University, Umeå, Sweden; h Danish Research Centre for Magnetic Resonance, Centre for Functional and Diagnostic Imaging and Research, Copenhagen University Hospital Hvidovre, Denmark; i Department of Radiation Sciences, Umeå University, Sweden; j Institute of Sports Medicine Copenhagen (ISMC), Copenhagen University Hospital Bispebjerg, Copenhagen, Denmark

**Keywords:** Burnout, Exhaustion disorder, Mental fatigue, Striatum, Working memory

## Abstract

Emerging evidence suggests that mental fatigue is a central component of the cognitive and clinical characteristics of stress-related exhaustion disorder (ED). Yet, the underlying mechanisms of mental fatigue in this patient group are poorly understood. The aim of this study was to investigate cortical and subcortical structural neural correlates of mental fatigue in patients with ED, and to explore the association between mental fatigue and cognitive functioning. Fifty-five patients with clinical ED diagnosis underwent magnetic resonance imaging. Mental fatigue was assessed using the Concentration subscale from the Checklist Individual Strength. Patients with high levels of mental fatigue (*n* = 30) had smaller caudate and putamen volumes compared to patients with low-moderate levels of mental fatigue (*n* = 25). No statistically significant differences in cortical thickness were observed between the groups. Mediation analysis showed that mental fatigue mediated the relationship between caudate volume and working memory; specifically, smaller caudate volume was associated with higher level of mental fatigue and mental fatigue was positively associated with working memory performance. Our findings demonstrate that the structural integrity of the striatum is of relevance for the subjective perception of mental fatigue in ED, while also highlighting the complex relationship between mental fatigue, cognitive performance and its neural underpinnings.

## Highlights

• Structural correlates of mental fatigue investigated in stress-related exhaustion.
• Patients were divided into high and low-moderate mental fatigue group.
• Patients with high mental fatigue had smaller caudate and putamen volumes.
• No significant differences in cortical thickness between the groups.
• Mental fatigue mediated the relationship between caudate volume and working memory.

## 1 Introduction

Stress-related exhaustion disorder (ED) is a clinical condition characterized by psychological and physical symptoms of exhaustion developed in response to long-term psychosocial stress (Grossi et al., 2015). ED has been associated with impaired cognitive performance, most consistently demonstrated in the domains of executive function, working memory, attention and processing speed (Ellbin et al., 2018, Jonsdottir et al., 2013, Krabbe et al., 2017, Oosterholt et al., 2012, Öhman et al., 2007). Impaired cognitive performance (Eskildsen et al., 2016, Jonsdottir et al., 2017) and elevated levels of subjective cognitive complaints (Eskildsen et al., 2016) can persist several years post rehabilitation, indicating that cognitive deficits can be a significantly debilitating and long-lasting symptom which constitute an important interventional target.

Fatigue is a multidimensional phenomenon with physical, emotional, behavioral and cognitive components (Chaudhuri and Behan, 2004). In stress-related ED, fatigue is a central clinical characteristic; a significant lack of psychological energy is part of the diagnostic criteria (Grossi et al., 2015), patients report high levels of fatigue (van Dam et al., 2011), and both physical and mental fatigue are described as residual symptoms by a large proportion of patients several years after treatment (Glise et al., 2020, Stenlund et al., 2012). Yet, despite its multifaceted nature and high prevalence in ED, the different aspects of fatigue have not been explicitly examined in this patient group. In the context of ED-related cognitive impairments, mental fatigue may be of particular relevance. Mental fatigue occurs across a wide range of clinical conditions, and is characterized by mental exhaustion and increased time needed for recovery following prolonged cognitive activity (Johansson et al., 2009). In ED, several studies have demonstrated that mental fatigue is evident when patients are performing effortful, cognitively demanding tasks. For example, Krabbe et al. (2017) found that, in conjunction with impaired performance on executive function and complex attention tasks, patients with ED experienced a marked increase in mental tiredness during and after cognitive testing. In a study focusing on patients with clinical burnout, Oosterholt et al. (2014) demonstrated that despite showing a relatively mild cognitive deficit, patients perceived performing the cognitive tests as effortful and fatiguing. Similar results have also been reported by van Dam et al. (2011) who found that, compared to healthy controls, burnout patients experienced elevated levels of fatigue, effort and aversion when conducting an attentional task, and that higher level of fatigue was associated with impaired task performance. Yet, despite the potential clinical relevance of mental fatigue in ED, the phenomenon has not been well described, and little is known about its underlying mechanisms.

Previous neuroimaging studies have demonstrated that, compared to healthy controls, patients with ED show reduced volumes in the caudate (Blix et al., 2013, Savic, 2015) and putamen (Blix et al., 2013), and larger amygdala volumes (Savic, 2015). Additionally, reduced gray matter volumes of the anterior cingulate cortex (ACC) and the dorsolateral prefrontal cortex (PFC) have been observed (Blix et al., 2013), as well as reduced cortical thickness in the medial PFC (Savic, 2015). Functional neural changes have also been reported, including reduced functional connectivity between the amygdala and the PFC, ACC and motor cortex (Golkar et al., 2014, Jovanovic et al., 2011), and altered prefrontal (Sandström et al., 2012) and fronto-striatal (Gavelin et al., 2017) neural activation during working memory processing. Importantly, these observations align with neuroanatomical models of mental fatigue, which suggest that the phenomenon is characterized by dysfunction in the subcortical-cortical network connecting the striatum and the PFC, including the striatum, the ACC, the dorsolateral and ventromedial PFC (Chaudhuri and Behan, 2000, Dobryakova et al., 2013). This model has been supported by structural and functional neuroimaging studies across different clinical groups in which fatigue is prevalent, such as in Parkinson’s disease (Kluger et al., 2019), multiple sclerosis (Genova et al., 2013) and traumatic brain injury (Kohl et al., 2009), but has yet to be empirically examined in ED.

The aim of this study was to use structural magnetic resonance imaging (MRI) to investigate cortical and subcortical structural correlates of mental fatigue in patients with ED. A further aim was to investigate the association between mental fatigue and cognitive functions that rely on the integrity of the fronto-striatal circuitry, namely executive function and working memory (Eriksson et al., 2015). We hypothesized that higher level of mental fatigue would be associated with smaller striatal volumes, reduced prefrontal and ACC cortical thickness, and impaired cognitive performance.

## 2 Methods

### 2.1 Participants and procedures

The study was conducted in accordance with the Declaration of Helsinki and approved by the Umeå Regional Ethical Review Board (Dnr 2010-53-31). All participants provided written informed consent prior to the start of the study. Participants were recruited as part of a randomized clinical trial which has previously been described in detail (Gavelin et al., 2018). Briefly, the trial investigated the effects of cognitive and aerobic training, offered as additions to a multimodal stress rehabilitation program, on cognitive function, psychological health and work ability in patients with ED. All participants fulfilled diagnostic criteria for ED, as outlined in the Swedish version of the International Classification of Diseases and Related Health Problems (ICD-10, code F43.8A) and were diagnosed by a psychologist and a physician. The diagnostic criteria for ED comprise experiencing psychological and physical exhaustion developed as a consequence of identifiable stressors, either work or non-work related, that have been present for at least six months duration (Grossi et al., 2015). The inclusion criteria for the randomized clinical trial were (1) confirmed diagnosis of ED; (2) 18–60 years old; (3) currently employed; (4) considered by a physician and a psychologist to be suitable for group-based stress rehabilitation; (5) no known abuse of alcohol or drugs; (6) not in the need of other treatment; and (7) not participating in other interventional study. Patients with other significant diagnoses in addition to ED (e.g. neurological or chronic psychiatric diagnoses), that required special care and adjustments, were not considered eligible for the standardized group-based stress rehabilitation, and consequently they were not included in this study. All participants took part in a 24-week stress rehabilitation program. As a part of the procedures of the overall interventional trial, after 12 weeks of stress rehabilitation, participants were randomized to one of three conditions: continued stress rehabilitation with (1) additional cognitive training, (2) additional aerobic training or (3) no additional training. The MRI was conducted before randomization, after 12 weeks of stress rehabilitation. At this time point, participants also completed a neuropsychological test session and self-report forms assessing psychological health. In total, 60 participants conducted MRI. Five participants were excluded from the analysis, two due to missing data from the MRI scanning and three due to missing behavioral data; thus the final sample consisted of 55 participants.

### 2.2 Measures

#### 2.2.1 Fatigue severity

While there is currently no gold standard for assessing fatigue in general (Dittner et al., 2004, Worm-Smeitink et al., 2017) and mental fatigue in particular (Kluger et al., 2013), it has been suggested that the use of a multidimensional scale is appropriate when exploring underlying mechanisms and specific aspects of fatigue (Dittner et al., 2004). One such instrument is the Checklist Individual Strength (CIS), (Beurskens et al., 2000) which was used in the present study to assess fatigue severity. This instrument consists of 20 items assessed on a 7-point Likert scale (1 = “Yes, that is true”, 7 = “No, that is not true”). It assesses four dimensions of fatigue: Subjective experience of fatigue (eight items, e.g., “I feel tired”), Activity (three items, e.g., “I don’t do much during the day”), Motivation (four items, e.g., “I feel no desire to do anything”), and Concentration (five items, e.g., “Thinking requires effort”). A total score was calculated for each domain, with higher score indicating more fatigue. We used the median of the Concentration subscale based on the norm scores for patients with chronic fatigue syndrome presented by Worm-Smeitink et al. (2017) to identify individuals with high levels of mental fatigue, whereby a score of ≥29 out of 35 was identified as high and a score of <29 was identified as low-moderate mental fatigue. The Concentration subscale shows moderate correlation with other measures of mental fatigue (Worm-Smeitink et al., 2017) and the normative scores presented by Worm-Smeitink et al. (2017) are based on a fairly large cohort of individuals with chronic fatigue syndrome (*n* = 1407), a patient group sharing clinical characteristics with ED (Maroti and Bileviciute-Ljungar, 2018), thus providing a reasonable approximation of the distribution of mental fatigue also for patients with stress-related ED.

#### 2.2.2 Cognitive function

The cognitive tests used in the Rehabilitation for Improved Cognition study has previously been described in detail (Gavelin et al., 2015). Briefly, three tests were used to assess executive function. The *n*-back task was used as a measure of updating ability (Kirchner, 1958). Participants were presented with lists of single digits (1–9) and asked to report whether the presented digit matched the digit presented one, two or three steps back. Number of correct responses minus number of false alarms in the 3-back condition was used as dependent measure. The Trail Making Test from the Delis-Kaplan Executive Functioning System was used as a measure of shifting ability, indexed by the time taken in seconds to complete the shifting condition as compared to connecting numbers (Delis et al., 2001). Finally, the Color-Word Interference Test from the Delis-Kaplan Executive Functioning System was used to assess inhibition (Delis et al., 2001). Time taken in seconds to complete incongruent trials as compared to time taken to read color words was used as a measure of inhibition cost. To assess working memory, we used number of correctly recalled sequences from Digit span forwards and backwards from WAIS-R (Wechsler, 1981) and Letter-number sequencing from WAIS-III (Wechsler, 1997). Finally, to assess verbal ability, participants conducted SRB:1, a 30-item multiple-choice synonym test (Dureman et al., 1971).

#### 2.2.3 Psychological health

The Shirom-Melamed Burnout Questionnaire (SMBQ) was used to assess levels of burnout (Melamed et al., 1992). This measure consists of 22 items rated on a 7-point Likert scale ranging from 1 (“almost never”) to 7 (“almost always”). The Hospital Anxiety and Depression Scale (HAD) was used to assess symptoms of anxiety and depression (Zigmond and Snaith, 1983). The scale consists of 14 items, seven targeting anxiety and depression respectively, rated on a four-point Likert scale (0–3). The Perceived stress questionnaire (PSQ) was used to measure level of perceived stress (Levenstein et al., 1993). This 30-item questionnaire is rated on a four-point Likert scale (1–4), with higher score indicating higher level of perceived stress.

### 2.3 MRI data acquisition and preprocessing

Structural MRI data was acquired on a 3T General Electric scanner equipped with a 32-channel head coil. High resolution T1-weighted images were collected using a 3D fast spoiled gradient echo sequence covering 180 slices with 1 mm thickness. Acquisition details included: TR = 8.2 ms; TE = 3.2 ms; flip angle 12°; field of view 25 × 25 cm. Freesurfer version 5.3 was used for brain segmentation. For the subcortical measurements volume (mm^3^) was used, and for the cortical segmentation cortical thickness (mm) from the Destrieux atlas (Destrieux et al., 2010). Regions of interests (ROI) were defined as areas in which structural alterations have been observed in patients with ED, as compared to healthy controls, based on previous studies (Blix et al., 2013, Savic, 2015, Savic et al., 2018), while also being outlined in neuroanatomical models of mental fatigue (Chaudhuri and Behan, 2000, Dobryakova et al., 2013). For the subcortical segmentations, we extracted the bilateral caudate and putamen volumes. For cortical thickness, we combined the bilateral superior and middle frontal gyri to produce a dorsolateral PFC ROI; the bilateral orbital gyri and sulci and gyrus rectus to produce a ventromedial PFC ROI; and the anterior and middle-anterior cingulate gyri and sulci to produce an ACC ROI.

### 2.4 Statistical analysis

Statistical analyses were performed using IBM SPSS Statistics version 26. All cognitive tests were *z*-transformed by standardizing them to the baseline mean and standard deviation, with higher score indicating better performance. A composite was created for working memory and executive functions, by averaging the *z*-scores for the three tests included in each domain. For working memory, one participant scored >3 standard deviations above the mean: this value was replaced with a score of 3 standard deviations above the mean.

Differences between the high and low-moderate fatigue group in background characteristics, clinical variables and cognitive function were investigated using independent samples *t*-test for continuous variables and Chi-square tests for categorical variables. Analysis of covariance (ANCOVA) was used to investigate group differences in subcortical volumes and cortical thickness, using age as a covariate. The association between mental fatigue and subcortical volumes, cortical thickness and cognitive performance was investigated using partial correlations, controlling for age. As a control analysis, PSQ and HAD depression score were also entered as covariates in the ANCOVA’s and correlation analyses. For the subcortical volumes, all analyses were conducted on the ratio between the volume of interest and the total intracranial volume. The caudate and putamen volumes had positively skewed distributions and were therefore log-transformed to conform more closely to normal distribution.

Finally, mediation analyses were performed using the lavaan package in R (Rosseel, 2012), testing whether mental fatigue mediated the relationship between structural neural integrity and cognitive performance. The mediation analysis focused specifically on the caudate, due to this region’s importance for higher-order cognitive functioning on the one hand (Grahn et al., 2008), and mental fatigue on the other hand. The confidence limits of the mediated (indirect) effect were generated using bootstrap resampling (*n* = 10,000) and the mediated effect was considered statistically significant if the confidence interval did not contain zero (MacKinnon et al., 2007). Age was included as a covariate in the model.

## 3 Results

### 3.1 Characteristics of high and low-moderate mental fatigue groups

Thirty patients (55%) were classified as having high levels of mental fatigue. The demographic, clinical and cognitive characteristics of the high and the low-moderate mental fatigue groups are shown in Table 1. The groups were similar in age, sex distribution, education level and verbal ability. The high mental fatigue group showed higher levels of burnout (Cohen’s *d* = 0.97) and perceived stress (Cohen’s *d* = 0.62) than the low-moderate mental fatigue group. No significant differences in levels of depression (Cohen’s *d* = 0.48) or anxiety (Cohen’s *d* = 0.20) were found. For the different subscales of CIS, patients in the high mental fatigue group showed a tendency towards higher levels of general subjective experience of fatigue (Cohen’s *d* = 0.54). No statistically significant difference in activity levels or reduced motivation was found between the groups, nor were there any statistically significant differences between the groups in executive function or working memory performance (Cohen’s *d* = 0.32 and 0.23 respectively, favoring the high mental fatigue group).

**Table 1:** Demographic, Clinical and Cognitive Characteristics.

Measure	High mental fatigue (*n* = 30)	Low-moderate mental fatigue (*n* = 25)	Statistics	Cohen’s *d*
Age	42.50 (7.76)	43.48 (9.89)	t(53) = 0.41, *p* = 0.68	

Sex, *n (%)*				
Female	24 (80%)	22 (88%)	χ^2^(1) = 0.64, *p* = 0.43	

Education*, n (%)*				
University	18 (60%)	16 (64%)	χ^2^(1) = 0.09, *p* = 0.76	
Verbal ability	22.43 (3.53)	22.52 (4.42)	t(53) = 0.08, *p* = 0.94	
Total intracranial volume (mm^3^)	1,509,209 (148180)	1,497,362 (190851)	t(53) = 0.26, *p* = 0.80	
SMBQ	5.31 (0.65)	4.55 (0.88)	t(53) = 3.65, *p* < 0.001	0.97
PSQ	78.87 (13.42)	70.28 (14.35)	t(53) = 2.29, *p* = 0.03	0.62
HAD Depression	7.90 (3.93)	6.16 (3.24)	t(53) = 1.77, *p* = 0.08	0.48
HAD Anxiety	10.23 (3.84)	9.48 (3.82)	t(53) = 0.73, *p* = 0.47	0.20
CIS Concentration	32.17 (2.25)	25.08 (3.30)	t(53) = 9.43, *p* < 0.001	2.51
CIS Subjective fatigue	42.73 (9.05)	37.72 (9.36)	t(53) = 2.01, *p* = 0.05	0.54
CIS Activity	13.20 (4.14)	12.32 (3.54)	t(53) = 0.84, *p* = 0.41	0.23
CIS Motivation	18.40 (3.28)	18.20 (3.34)	t(53) = 0.22, *p* = 0.82	0.06
Executive function	0.14 (0.65)	−0.09 (0.76)	t(52) = 1.19, *p* = 0.24	0.32
3-back	23.24 (6.79)	20.12 (8.99)		
Shift cost^a^	47.73 (26.49)	50.00 (24.22)		
Inhibition cost^a^	28.97 (12.03)	31.20 (10.64)		
Working memory	0.10 (0.91)	−0.13 (1.07)	t(53) = 0.86, *p* = 0.39	0.23
Digit span forward	7.27 (2.41)	6.84 (1.84)		
Digit span backward	6.53 (1.70)	6.56 (2.50)		
Letter-number sequencing	10.33 (2.19)	9.48 (2.57)		

*Note.* Data is shown as mean (standard deviation) unless otherwise indicated. Cognitive constructs are reported as *z*-scores. Cohen’s *d* is calculated as (M_high_ − M_low-moderate_)/√[(SD_high_^2^ + SD_low-moderate_^2^)/2]. SMBQ = Shirom-Melamed Burnout Questionnaire. PSQ = Perceived Stress Questionnaire. HAD = Hospital Anxiety and Depression Scale. CIS = Checklist Individual Strength.

a Lower score indicates better performance.

### 3.2 Group differences in cortical thickness and subcortical volumes

**Fig. 1:**
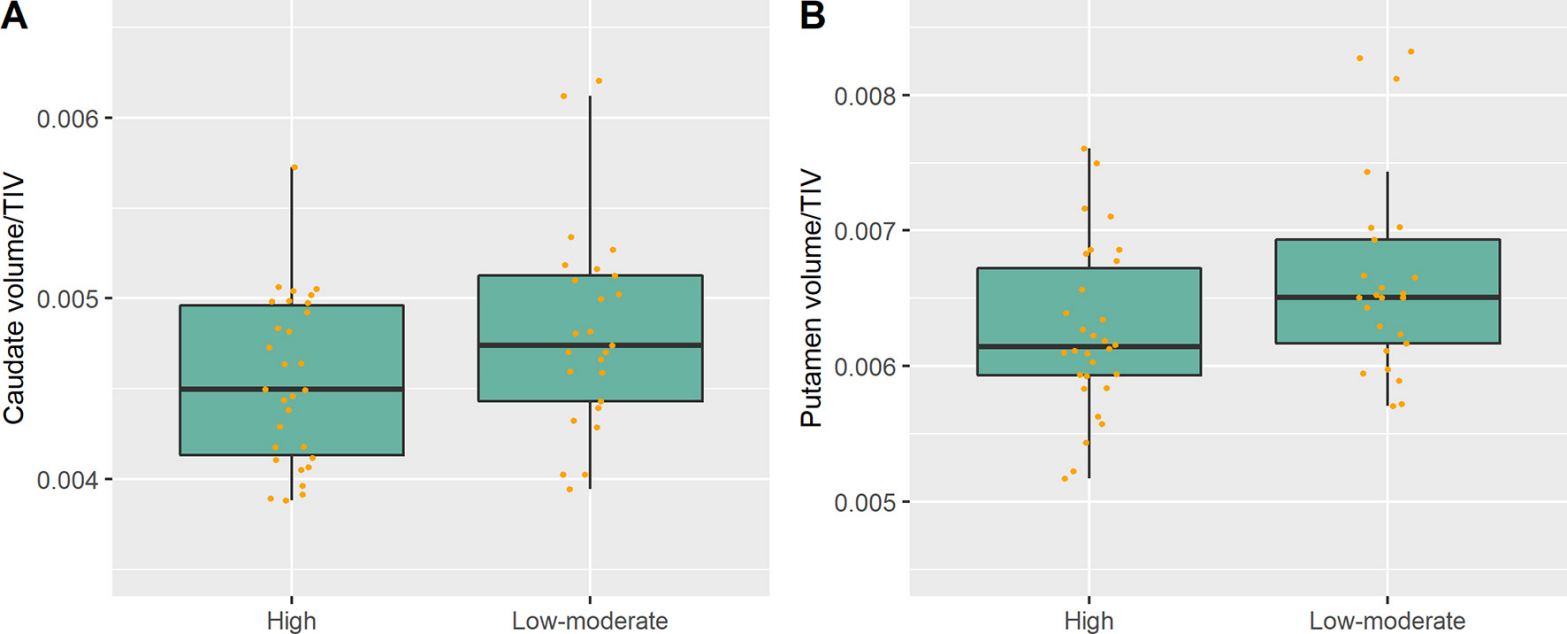
Group differences in subcortical volumes. Ratio of (a) caudate and (b) putamen volume relative to the total intracranial volume in the high and low-moderate mental fatigue group, respectively. TIV = total intracranial volume.

Group means and standard deviations for cortical thickness and subcortical volumes are shown in Table 2. Patients in the high mental fatigue group showed significantly smaller caudate and putamen volumes relative to the total intracranial volumes than patients with low-moderate mental fatigue (Fig. 1). The difference between the groups in caudate and putamen volumes remained statistically significant also after controlling for level of perceived stress and depression (*p* = 0.04 and *p* = 0.03, for caudate and putamen respectively). No statistically significant differences in cortical thickness were found between the groups.

**Table 2:** Subcortical Volumes and Cortical Thickness in the Regions of Interest for the High and Low-Moderate Mental Fatigue Groups.

Volume	High mental fatigue (*n* = 30)	Low-moderate mental fatigue (*n* = 25)	Statistics
Caudate (mm^3^)	6841.23 (833.95)	7205.88 (1155.92)	F(1, 52) = 4.99, *p* = 0.03^a^
Putamen (mm^3^)	9445.66 (1322.49)	9931.94 (1672.33)	F(1, 52) = 5.36, *p* = 0.03^a^
dlPFC (mm)	2.71 (0.11)	2.73 (0.13)	F(1, 52) = 1.11, *p* = 0.30^b^
vmPFC (mm)	2.55 (0.09)	2.54 (0.09)	F(1, 52) = 0.42, *p* = 0.52^b^
ACC (mm) c	2.61 (0.10)	2.60 (0.10)	F(1, 49) = 0.004, *p* = 0.95^b^

*Note*. Data is shown as mean (standard deviation). dlPFC = dorsolateral prefrontal cortex. vmPFC = ventromedial prefrontal cortex. ACC = anterior cingulate cortex.

a Analysis of covariance controlling for age, based on log transformed ratios of the respective structural volume and the total intracranial volume.

b Analysis of covariance controlling for age.

c *n* = 52

### 3.3 Associations between mental fatigue, brain structure and cognitive performance

**Fig. 2:**
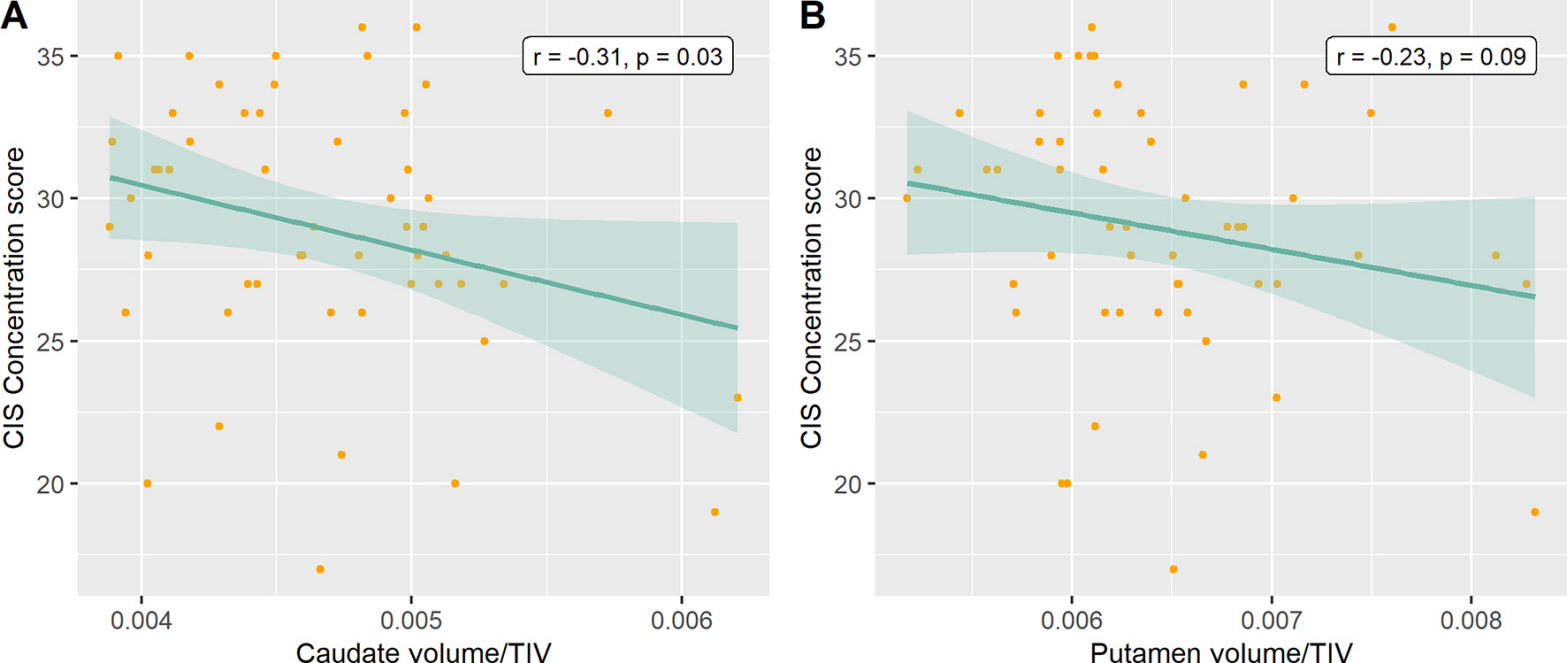
Associations between mental fatigue and subcortical volumes. Scatterplot of the correlation between CIS Concentration score and the ratio of (a) caudate volume and (b) putamen volume relative to the total intracranial volume. CIS = Checklist Individual Strength. TIV = total intracranial volume.

Results showed a negative correlation between CIS Concentration score and caudate volumes, indicating that smaller caudate volumes were associated with higher levels of mental fatigue (*r* = −0.31, *p* = 0.03, Table 3). The correlation remained significant when controlling for level of perceived stress and depression (*r* = −0.30, *p* = 0.03). No other statistically significant correlations were found. Fig. 2 shows the association between mental fatigue and the subcortical volumes.

**Table 3:** Correlation Between Mental Fatigue, Regions of Interest and Cognitive Performance.

	CIS Concentration score^a^
Caudate volume	*r* = −0.31, *p* = 0.03
Putamen volume	*r* = −0.23, *p* = 0.09
dlPFC cortical thickness	*r* = −0.17, *p* = 0.22
vmPFC cortical thickness	*r* = 0.04, *p* = 0.78
ACC cortical thickness	*r* = -0.06, *p* = 0.68
Executive function	*r* = 0.10, *p* = 0.46
Working memory	*r* = 0.23, *p* = 0.10

*Note*. CIS = Checklist Individual Strength. dlPFC = dorsolateral prefrontal cortex. vmPFC = ventromedial prefrontal cortex. ACC = anterior cingulate cortex.

a Partial correlation, controlling for age.

### 3.4 Mediation analysis

**Fig. 3:**
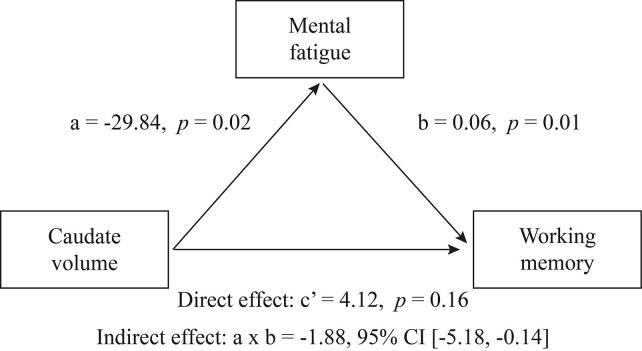
Mediation model. Mental fatigue mediated the relationship between caudate volume and working memory performance, such that smaller caudate volume was associated with higher level of mental fatigue and better working memory performance.

Due to the very weak association found between mental fatigue and executive function in the correlation analysis, the mediation model was restricted to working memory as the cognitive outcome. The mediation analysis showed that mental fatigue mediated the relationship between caudate volume and working memory performance (a × b path, indirect effect, unstandardized coefficient = −1.88, 95% CI [−5.18, −0.14]). Fig. 3 shows the mediation model. The indirect effect was negative, indicating that smaller caudate volume was associated with higher level of mental fatigue; and that mental fatigue was positively associated with working memory performance. In contrast, the direct effect was positive, suggesting that smaller caudate volume predicted worse working memory performance, however, this path was not statistically significant (c’ path, unstandardized coefficient = 4.12, *p* = 0.16).

## 4 Discussion

The aim of this study was to investigate cortical and subcortical structural correlates of mental fatigue in patients with stress-related ED. Furthermore, we investigated the association between mental fatigue and executive function and working memory performance. We found that patients with high levels of mental fatigue had smaller volumes in the caudate and putamen compared to patients with low-moderate mental fatigue, and that the level of mental fatigue was negatively associated with caudate volume. We found no structural correlates of mental fatigue in the dorsolateral PFC, the ventromedial PFC or the ACC. Furthermore, an indirect negative effect of caudate volume on working memory performance was found, mediated by mental fatigue.

Our finding of an association between mental fatigue and structural volumes of the caudate and the putamen aligns with Chaudhuri and Behan (2000) influential model on central fatigue. According to this framework, mental fatigue is characterized by difficulties in initiating and maintaining performance on cognitive tasks requiring self-motivation, due to dysfunction in the fronto-striatal circuitry involved in motivational processes important for internal drive (Chaudhuri and Behan, 2000). This model has been supported by neuroimaging studies in several clinical groups in which fatigue is a prominent symptom; specifically, fatigue has been associated with reduced gray matter volumes and lesions in the striatum in Parkinson’s disease (Kluger et al., 2019), multiple sclerosis (Damasceno et al., 2016) and stroke (Tang et al., 2013). Our results also converge with prior research that have demonstrated reduced volumes of the caudate and the putamen in patients with ED as compared to healthy controls (Blix et al., 2013, Savic, 2015, Savic et al., 2018). Here, we extend those findings by demonstrating an association between the integrity of striatal regions and mental fatigue in the patient group. The correlation analyses showed that this association was stronger for caudate than putamen volume, which is in line with the involvement of the caudate in cognitive processing (Grahn et al., 2008). Although prior research has also demonstrated reduced cortical thickness and gray matter volumes in the medial and dorsolateral PFC and the ACC in ED (Blix et al., 2013, Savic, 2015), we did not find any relationship between cortical thickness in these regions and mental fatigue. These observations converge with a recent study showing that fatigue was associated with gray matter volumes of the caudate and putamen, but not dorsolateral PFC and ACC, in patients with Parkinson’s disease (Kluger et al., 2019). In patients with ED, structural and functional alterations in the PFC and the ACC have been associated with impaired ability to down-regulate negative emotion (Golkar et al., 2014, Savic et al., 2018), and thus the integrity of these regions may be of relevance for stress regulation in the patient group, rather than mental fatigue.

We found no significant relationship between mental fatigue and cognitive performance in the correlation analyses. However, in the mediation analysis, there was a positive association between mental fatigue and working memory performance (as indicated by the statistically significant b-path, see Fig. 3). Furthermore, the mediation model suggested an indirect negative effect of caudate volume on working memory performance through the mediating pathway of mental fatigue. That is, smaller caudate volume was associated with higher levels of mental fatigue and better working memory performance. Although somewhat counter intuitive, these results could perhaps be understood if viewing mental fatigue as reflective of the mental effort required to perform cognitive tasks. It has previously been hypothesized that ED is characterized by a high effort approach; that is, that patients with ED can compensate for cognitive impairment through increased effort (Krabbe et al., 2017, Oosterholt et al., 2014, van Dam et al., 2015), potentially by a compensatory recruitment of fronto-striatal neural resources during task performance (Gavelin et al., 2017). The results from the mediation model lends some support to this hypothesis and suggest that the relationship between caudate volume and working memory performance may be modulated by compensatory effort and increased fatigue. Importantly, the direct and indirect effect were of opposite signs; thus, while smaller caudate volume was associated with worse working memory performance (positive direct effect), this association may in part be counteracted by compensatory effort, reflected by higher level of mental fatigue (negative indirect effect; see Zhao et al., 2010 for a review). Although the non-significant direct effect warrants caution when interpreting these findings, this nevertheless fits well with clinical observations that patients with ED can uphold cognitive task performance for a limited period of time (e.g., in neuropsychological testing), but that it comes at a large cost. It also aligns with previous studies demonstrating that patients with ED generally show small-moderate impairment in standardized cognitive tests, while reporting substantial cognitive problems in everyday life (Krabbe et al., 2017, Oosterholt et al., 2014, Oosterholt et al., 2012). Since everyday life usually requires more sustained cognitive performance, compensatory strategies may be ineffective. Researchers have previously argued for the importance of distinguishing between subjective perceptions of mental fatigue and cognitive fatigability, i.e., decreased performance following sustained cognitive effort, which are distinct and potentially independent phenomena (Kluger et al., 2013). Our findings highlight the complex relation between mental fatigue, cognitive performance and its neural underpinnings, and an important avenue for future research is to explore the association between mental fatigue and performance fatigability in ED.

Given the close link between mental fatigue and motivational processes, it is interesting to note that no differences in ratings of reduced motivation were found between the high and the low-moderate mental fatigue groups. While it might be expected that a decrease in motivation would be exacerbated in the high fatigue group, our results nevertheless align with observations that patients with ED are motivated to invest effort into performing cognitive tasks, despite experiencing high levels of mental fatigue; this clinical observation has also been confirmed in a prior study (Oosterholt et al., 2014). On the other hand, patients with ED show impaired responsiveness to reward (van Dam et al., 2011). Recent frameworks of mental fatigue suggest that perceptions of fatigue arise as a result of an effort-reward imbalance (Boksem and Tops, 2008, Dobryakova et al., 2013); thus, this trans-diagnostic symptom may be the consequence of altered reward processing in clinical populations (Dobryakova et al., 2013), with a possible underlying role of the dopaminergic system (Dobryakova et al., 2015). Dopamine is also involved in updating of working memory representations in the PFC (O'Reilly, 2006) and deficits in motivational and reward processes have been suggested as a potential mechanism for the cognitive control deficits seen in depression (Grahek et al., 2018, Grahek et al., 2019). Future studies should investigate mental fatigue from the perspective of effort and reward and the relevance of physiological alterations in the motivational system in patients with ED, as well as its relation to cognitive functioning.

Some limitations of this study need to be addressed. First, this study had no control group and thus we are unable to determine whether the participants in our study show reduced subcortical volumes and impaired cognitive performance in relation to healthy individuals. However, the ROIs were chosen based on previous studies which have demonstrated structural alterations in these specific regions in patients with ED as compared to healthy controls, and the results from our study provide novel insights into the associations between the structural neural integrity of these regions and mental fatigue. Nevertheless, more research is needed to explore whether these associations are the same in individuals with and without ED. In an additional control analysis, we used hippocampal volume as a control region and found no significant difference between the high and the low-moderate mental fatigue groups (*p* = 0.60). Second, the high mental fatigue group also showed higher levels of burnout and perceived stress than the group with low-moderate mental fatigue. Given that “a significant lack of psychological energy” is part of the diagnostic criteria for ED (Grossi et al., 2015), it is hardly surprising that mental fatigue is not an isolated phenomenon in this patient group, but rather an integral part of the clinical picture. We chose not to control for burnout since the SMBQ also includes items relating to mental fatigue. However, our results were robust when controlling for levels of perceived stress and depression, suggesting that mental fatigue has explanatory value over and above general psychopathology. Third, in line with previous observations that women are over-represented in ED (Glise et al., 2012), the majority of the participants in our study were female. The small number of male participants prevented us from exploring potential gender differences, which may be important since previous research has suggested that neural alterations in stress-related exhaustion are more pronounced in women (Savic, 2020, Savic et al., 2018). Whether the neural correlates of mental fatigue in ED differ between men and women should therefore be explored in future studies. On a final note, our study focused specifically on executive function and working memory, and information on patients’ cognitive performance in other domains, such as episodic memory and processing speed, and its relation to mental fatigue could also be of value.

## 5 Conclusions

In conclusion, this study is the first to empirically investigate Chaudhuri and Behan (2000) model on central fatigue in patients with clinical ED diagnosis and show that the structural integrity of the striatum is of relevance for the subjective perception on mental fatigue in this patient group. Our findings highlight the importance of considering mental fatigue when studying the cognitive impairments that are a prevalent and long-lasting symptom in ED. We provide some important directions for future research, including investigating whether patients’ perception of mental fatigue is also reflected by increased cognitive fatigability and exploring altered reward processing as an underlying mechanism.

## CRediT authorship contribution statement

**Hanna Malmberg Gavelin:** Conceptualization, Methodology, Formal analysis, Visualization, Writing - original draft, Writing - review & editing. **Anna Stigsdotter Neely:** Conceptualization, Methodology, Writing - review & editing, Funding acquisition. **Tora Dunås:** Formal analysis, Writing - review & editing. **Therese Eskilsson:** Conceptualization, Methodology, Writing - review & editing. **Lisbeth Slunga Järvholm:** Conceptualization, Methodology, Writing - review & editing, Funding acquisition. **Carl-Johan Boraxbekk:** Conceptualization, Methodology, Formal analysis, Writing - review & editing, Supervision.

## Declaration of Competing Interest

The authors declare that they have no known competing financial interests or personal relationships that could have appeared to influence the work reported in this paper.
